# Production of silk sericin/silk fibroin blend nanofibers

**DOI:** 10.1186/1556-276X-6-510

**Published:** 2011-08-25

**Authors:** Xianhua Zhang, Masuhiro Tsukada, Hideaki Morikawa, Kazuki Aojima, Guangyu Zhang, Mikihiko Miura

**Affiliations:** 1 Faculty of Textile Science and Technology, Shinshu University, 3-15-1 Tokida, Ueda City, Nagano 386-8567, Japan

**Keywords:** silk sericin, silk fibroin, electrospinning, nanofibers, structure

## Abstract

Silk sericin (SS)/silk fibroin (SF) blend nanofibers have been produced by electrospinning in a binary SS/SF trifluoroacetic acid (TFA) solution system, which was prepared by mixing 20 wt.% SS TFA solution and 10 wt.% SF TFA solution to give different compositions. The diameters of the SS/SF nanofibers ranged from 33 to 837 nm, and they showed a round cross section. The surface of the SS/SF nanofibers was smooth, and the fibers possessed a bead-free structure. The average diameters of the SS/SF (75/25, 50/50, and 25/75) blend nanofibers were much thicker than that of SS and SF nanofibers. The SS/SF (100/0, 75/25, and 50/50) blend nanofibers were easily dissolved in water, while the SS/SF (25/75 and 0/100) blend nanofibers could not be completely dissolved in water. The SS/SF blend nanofibers could not be completely dissolved in methanol. The SS/SF blend nanofibers were characterized by Fourier transform infrared (FTIR) spectroscopy, differential scanning calorimetry, and differential thermal analysis. FTIR showed that the SS/SF blend nanofibers possessed a random coil conformation and ß-sheet structure.

## Introduction

Electrospinning is an interesting and effective process for producing nanoscale fibers with diameters in the nanometer to micrometer range. These nanofibers have high functionality, high specific surface area, and high porosity with very small pore size [1,2]. Therefore, the micro/nanofibers can simulate the extracellular matrix and enhance cell migration and proliferation. They are applied in the biomedical domain for drug delivery, wound dressing, tissue engineering scaffolds, and other uses [3-6].

Silk cocoon filaments from the *Bombyx mori *silkworm are composed of SS and SF. SS is bio-synthesized exclusively in the middle silk gland and is about 25-30% of silk fiber mass. Sericin coats fibroin threads to glue them together and fills the gaps to enhance the toughness of the cocoon fiber [7]. Sericin is composed of a large number of hydrophilic amino acids, such as serine, glycine, lysine, etc. Sericin is an important bio-material, which can be used in the medical and cosmetic fields, because it shows good compatibility to human tissues, biodegradation and oxidation resistance, antibacterial properties, and UV resistance. Additionally, sericin absorbs and releases moisture readily and exhibits the inhibitory activity of tyrosine and kinase, and is used widely in medical applications [8,9]. SF has been widely used in tissue engineering because of specific functional properties, including excellent biocompatibility, good oxygen and water vapor permeability, and biodegradability [10-12]. There are large numbers of publications on SF and SF/polymer blend nanofibers [13,14], but there are few publications on the SS/SF blend nanofibers.

In this study, we present a novel way to prepare SS/SF nanofibers. Our results demonstrate the successful preparation of SS/SF blend nanofibers with smooth surfaces via electrospinning from SS/SF blend solutions, and we analyze their molecular conformation and physical properties.

## Experiment

### Materials

Sericin as the powder form of silk sericin (SS) was purchased from Wako Pure Chemical Industries, Ltd. (3-1-2, Chuoh-ku, Osaka city, 540-8605, Japan) (Lot. CDR4258). Silk fibroin (SF) film was prepared by casting 2 wt.% SF solution in flat-bottomed polystyrene dishes at room temperature in a flow cabinet. SF solution was prepared by dissolving SF fibers in 8 M LiBr solution at 60°C for 30 min and then dialyzed against water at 5°C for 4 days using cellulose dialysis tubes.

### Preparation of the SS/SF blended solution

SS solution (20 wt.%) and SF solution (10 wt.%) were prepared by stirring the samples in trifluoroacetic acid (TFA) at 25°C for 3 h. Solutions of 0.45, 0.3, and 0.15 g SS were mixed with 0.3, 0.6, and 0.9 g SF solutions, respectively, so that the SS/SF (*w*/*w*: 75/25, 50/50, and 25/75) blend solutions were prepared. The SS/SF (75/25) blend nanofibers were produced from solutions containing 75 wt.% SS and 25 wt.% SF. The pure and mixed solutions were stirred for 3 h. These sample solutions were stored in the refrigerator (4°C) for 12 h, while the electrospinning solution was prepared. The sample nanofibers were produced by electrospinning the SS/SF solutions, having different sample compositions (SS/SF: 100/0, 75/25, 50/50, 25/75, and 0/100).

### Electrospinning setup and process

Electrospinning of the solutions was conducted under normal atmospheric conditions. The electrospinning apparatus consisted of a syringe (SS-01T, Terumo Corporation, Tokyo, Japan), needle (NN-2238N, Terumo Corporation), aluminum collecting screen, and syringe pump. The spinning speed of the syringe pump was adjusted within the range 0.003-0.320 cm/min. High voltage power (Kato Tech Company, Tokyo, Japan), 26, Nishi 9-jou, Minami-ku, Kyoto city, 601-8447, Japan) was supplied in the range 0 to 40 kV. The sample solution was placed into the 1-ml syringe, with a 21-gauge needle (inner diameter 0.3 mm). The voltage was applied between the end of the needle and collecting screen. Nanofiber mats were produced on the aluminum collecting screen. The electrospinning was conducted with values of working distance, applied voltage, and flow rate at 15 cm, 25 kV, and 0.06 cm/min, respectively.

### Characterization

The morphology and the diameter of nanofibers were determined with a scanning electron microscope (SEM) (S-3000N, Hitachi, 1-6-6, Marunouchi, Chiyoda-ku, Tokyo, 100-8280, Japan. For SEM measurements, samples were placed on an aluminum circular plate and coated with a gold layer. The mean diameter and distribution were obtained using the commercial software package, SPSS.

Fourier transform infrared (FT-IR) spectroscopy was measured with a Shimadzu FT-IR-8400S infrared spectrometer (Shimadzu corporation, 1, Kuwabara-machi, Chuoh-ku, Kyoto City, 604-8511, Japan) by the ATR method in the region of 1800-700 cm^-1 ^at room temperature.

The spectra of samples were acquired in transmittance mode with a resolution of 4 cm^-1 ^and spectral range of 4,000-500 cm^-1^. Infrared spectra were recorded from 16 scans per sample.

Differential scanning calorimetry (DSC) curves of samples were measured with a DSC instrument (Thermo Plus DSC 8230, Rigaku Corporation, Tokyo, Japan) under normal atmospheric conditions, at a heating rate of 10°C/min. The temperature ranged from room temperature to 300°C, and the sample weight was 3 mg.

Differential thermal analysis (DTA) measurements were carried out using a DTA instrument (Thermo Plus TG 8120, Rigaku Corporation) under a nitrogen atmosphere in the range from room temperature to 300°C and at a heating rate of 10°C/min. The sample weight was 6 mg.

## Results and discussion

### SEM images and diameter distribution of the SS/SF blend nanofibers

**Figure 1:**
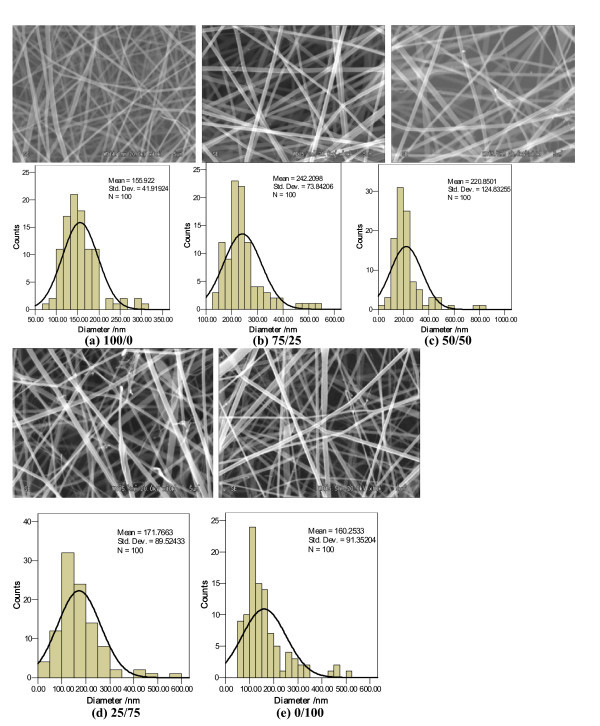
**SEM micrographs and diameter distributions of the SS/SF blend nanofibers**. SS/SF (*w*/*w*): (a) 100/0, (b) 75/25, (c) 50/50, (d) 25/75, (e) 0/100.

Figure 1 shows the SS/SF nanofibers and their corresponding diameter distributions for the different ratios of SS/SF. "As-spun" nanofibers exhibited smooth surfaces, round cross sections, and bead-free structures. The diameters ranged between 33 and 837 nm. The average diameters of the SS/SF blend nanofibers in Figure 1a,b,c,d,e were 156, 242, 221, 172, and 160 nm, respectively. The average diameters of the SS/SF (75/25, 50/50, and 25/75) blend nanofibers were thicker than SS (100/0) and SF (0/100) nanofibers. The average diameters of SS/SF blend nanofibers (Figure 1b,c,d) decreased with an increasing amount of the SF component.

### Effect of dissolution time

We prepared blend solutions by the following procedures. The SS/SF blend solution was firstly stirred at 25°C for 6 h, then stored in the refrigerator (4°C) for 12 h, and stirred again at 25°C for 222 h (10 days total). Immediately after stirring, sample nanofibers were produced by electrospinning.

**Figure 2:**
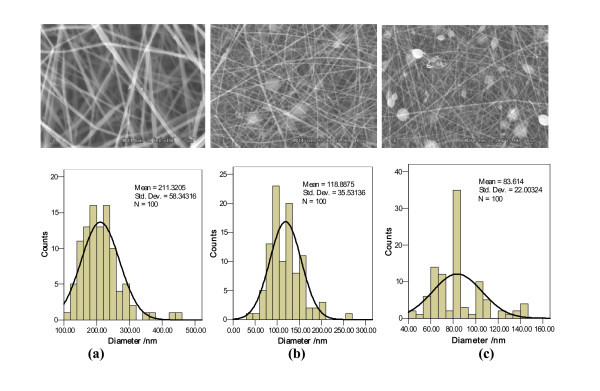
**SEM micrographs and diameter distributions of the SS/SF (50/50) blend nanofibers**. In relation to the dissolving time. Dissolving time and SS/SF (*w*/*w*): (a) 10 days, 100/0, (b) 10 days, 50/50, (c) 10 days, 0/100.

Figure 2 showed the SEM micrographs and diameter distribution diagrams of the SS, SS/SF (50/50), and SF nanofibers. SS nanofibers (Figure 2a) obtained by electrospinning with SS solution, having a dissolving time of 10 days, demonstrated bead-free and excellent nanofibers. The SS/SF (50/50) and SF nanofibers showed smooth nanofibers containing some circular and spindle-like beads. The number of beads in the SF nanofibers (Figure 2c) was much more than that in the SS/SF (50/50) (Figure 2b) blend nanofibers.

It is of important to note that the average diameter of nanofibers (100/0) is about 155 nm (Figure 1a), whereas being 211 nm in Figure 2a. This slight contradiction is probably due to the dissolving conditions, including dissolving temperature and time of the nanofibers. The dissolving temperature and time of SS/SF 100/0 (Figure 1a) are 25°C and 3 h, respectively, while dissolving temperature and time for the nanofibers from SS/SF 100/0 (Figure 2a) are 25°C and 10 days. We further research the effect of the dissolving conditions on diameter distribution of nanofibers. The average diameters of the SS/SF blend nanofibers (Figure 2a,b,c) were 211, 119, and 83 nm. The average diameters of these SS/SF nanofibers decreased with increasing SF content.

### Solubility of the SS/SF blend nanofibers

We examined the solubility of the SS/SF blend nanofibers in water and in methanol. The SS nanofibers were completely dissolved in water after a short immersion time (1 h), while the SF nanofibers were not dissolved in water. The SS/SF blend nanofibers, containing higher amounts of SS, were more easily dissolved in water. The SS/SF (100/0, 75/25, and 50/50) blend nanofibers were completely dissolved in water within 1 h. However, when the SF component was greater than the SS component, as in the SS/SF blend nanofibers (25/75), they could not be completely dissolved in water. Over the same time period, the SS/SF blend nanofibers could not be dissolved in methanol.

### FTIR spectra

**Figure 3:**
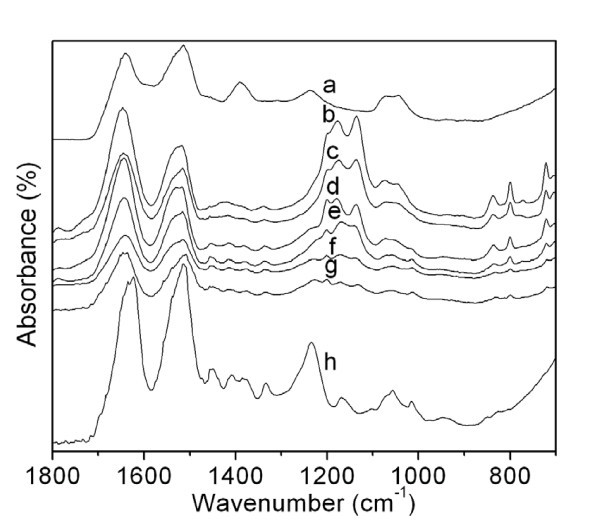
**FTIR spectra of SS, SS/SF blend nanofibers, heated SS/SF (50/50) blend nanofibers, and SF**. Sample: a, powder form of silk sericin; b, c, d, e, f, SS/SF blend nanofibers with different compositions (100/0, 75/25, 50/50, 25/75, 0/100); g SS/SF blend nanofiber (50/50), heat-treated at 160°C for 30 min; h SF film.

FTIR spectroscopy is a powerful technique for studying structure at the molecular level. FTIR shows the specific absorption bands sensitive to the molecular conformation of silk proteins [15]. The spectra of SS and the SS/SF blend nanofibers (Figure 3a,b,c,d,e,f) were characterized by the absorption bands at 1,641, 1,647, 1,645, 1,645, 1,643, and 1,643 cm^-1^, which were attributed to the random coil conformation. SF showed at 1,625 cm^-1 ^(amide I), and the SS/SF nanofibers showed absorption peaks at around 1,512 cm^-1 ^(amide II), attributed to the ß-sheet conformation. Amide III showed in SS and SF at 1,238 and 1,233 cm^-1 ^attributable to ß-sheet structure, while the absorption bands of amide III of the SS/SF (50/50, 25/75, and 0/100) blend nanofibers (Figure 3d,e,f) showed very small shoulder peaks. At the same time, amide III disappeared in the SS/SF (100/0 and 25/75) blend nanofibers' spectra (Figure 3b,c). It is worth noting that a shoulder absorption band at 1,218 cm^-1 ^exists in the TFA spectrum (not shown in Figure 3). The molecular conformation of the SS/SF blend nanofibers are composed of a random coil and ß-sheet structure (Figure 3).

The IR spectra of the SS/SF blend nanofibers (Figure 3b,c,d,e,f) were similar to each other. The minor absorption band at 1,787 cm^-1 ^did not appear in the SS or SF samples (Figure 3a,h). According to FTIR spectra of TFA, performed by FTIR analysis equipped with an ATR (attenuated total reflectance) accessory and ZnSe crystal, the absorption at 1,787 cm^-1 ^is specifically observed (unpublished data). However, according to Figure 3, it is difficult to observe this peak for curves d and f. It seems that absorption band at 1,787 cm^-1 ^is due to the trace amount of TFA contained in the SS/SF blend nanofibers and that the molecular interaction between the trace amount of TFA and SS is slightly stronger than that between TFA and SF.

It was of interest to note that this absorption band was detected in the SS/SF blend nanofibers (Figure 3b,c,d,e,f). The SS/SF (50/50, 25/75, and 0/100) blend nanofibers (Figure 3d,e,f) and SF (Figure 3h) possessed a minor absorption band at 1,454 cm^-1^. The SS/SF blend nanofibers and SF showed weak absorption bands at 1,408 and 1,334 cm^-1^. SS displayed a distinct absorption band at 1,390 cm^-1^, and SF showed a weak peak at 1,386 cm^-1^. However, this peak had disappeared in the SS/SF (100/0 and 75/25) blend nanofibers' spectra (Figure 3b,c). Around 1,200 cm^-1^, there was a weak peak exhibited in the spectra of the SS/SF blend nanofibers except for SS nanofibers. The SF (Figure 3h) showed weak absorption bands at 1,168 and 1,100 cm^-1^. All SS/SF blend nanofibers (Figure 3b,c,d,e,f) had the absorption bands around 1,170 and 1,136 cm^-1^. SS nanofibers possessed absorption bands around 1,072 and 1,047 cm^-1^, while SF nanofibers exhibited at around 1,014 cm^-1^. For these absorption bands, the SS/SF blend nanofibers showed absorption band characteristics of both SS and SF pure components overlapping in the corresponding absorption region. The SS/SF blend nanofibers showed absorption bands at 837, 801, and 720 cm^-1^. On the other hand, SS and SF did not have these absorption bands. The intensity of these absorption bands increased with increasing SS content. The intensity of the FTIR spectrum of SS/SF (50/50) blend nanofibers, heat-treated at 160°C for 30 min shown in Figure 3g, became minor compared with those of the SS/SF (50/50) nanofibers (Figure 3d).

### DSC curves

**Figure 4:**
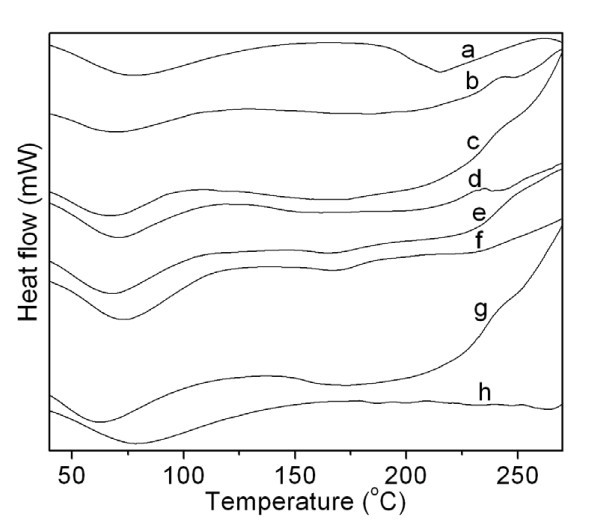
**DSC curves of SS, SS/SF blend nanofibers, SF, and heated SS/SF (50/50) blend nanofibers**. Sample abbreviation is the same in Figure 3.

In order to analyze the thermal behavior of the samples, DSC measurements were conducted. Figure 4 shows the DSC curves for SS, SS/SF blend nanofibers, SF, and the heated SS/SF (50/50) blend nanofibers. All the samples (a-h) showed endothermic peaks at around 60°C to 70°C, which were probably attributable to the evaporation of water or solvent remaining in the samples. In addition to this endothermic peak, SS showed a major endothermic peak at 215°C, due to the thermal decomposition of sericin, while SF showed small decomposition peaks at 187°C, 200°C, 264°C, 232°C, 248°C, and 218°C.

The SS/SF (100/0, 75/25, and 50/50) blend nanofibers (Figure 4b,c,d) showed broad decomposition peaks from 138°C to 215°C, 122°C to 203°C, and 138°C to 213°C, respectively. At the same time, weak new peaks at 248°C and 241°C occurred in the SS/SF (100/0 and 50/50) blend nanofibers (Figure 4b,d), respectively. With the SF component increasing up to 75% and 100%, the broad decomposition peaks of the blend nanofibers disappeared (Figure 4e,f). The SS/SF (25/75) showed only one small new decomposition peak at 165°C (Figure 4e). The SF (0/100) blend nanofibers (Figure 4f) showed two decomposition peaks at 168°C, which are new decomposition peaks, and 230°C attributed to SF (Figure 4h).

The SS/SF (50/50) blend nanofibers had a broad decomposition peak from 138°C to 213°C (Figure 4b), while the heated SS/SF (50/50) blend nanofibers showed the corresponding broad decomposition peak from 163°C to 181°C (Figure 4g). The small decomposition peak that appeared at 241°C for the SS/SF (50/50) blend nanofibers (Figure 4b) disappeared in the heated SS/SF (50/50) blend nanofibers (Figure 4g). It is assumed that the minor endothermic peak around 160°C is due to the removal of FTA, which contained quite slightly in the SS/SF nanofibers in the heating process, because the heat-treated SS/SF nanofiber does not show this endothermic peak.

### DTA analysis

**Figure 5:**
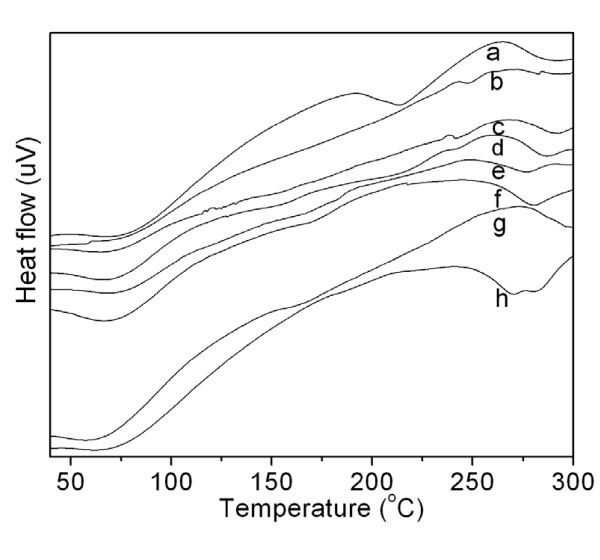
**DTA curves of SS, SS/SF blend nanofibers, SF, and heated SS/SF (50/50) blend nanofibers**. Sample abbreviation is the same in Figure 3.

All the samples showed endothermic peaks at around 60°C to 70°C that are due to the evaporation of water or solvent contained in the samples (Figure 5). The SS displayed two major endothermic peaks at 214°C and 288°C. SF (Figure 5h) showed two endothermic peaks in the temperature range of 270°C and 281°C. The SS/SF (100/0 and 75/25) blend nanofibers displayed an endothermic peak at around 240°C. The SS/SF blend nanofibers (Figure 5b,c,d,e,f) showed endothermic peaks at 283°C, 292°C, 287°C, 277°C, and 281°C, corresponding to the thermal decomposition of SF.

DTA curves of the SS/SF (50/50) (Figure 5d) and SS/SF (50/50) blend nanofibers, heat-treated at 160°C (Figure 5g), are shown in Figure 5. The prominent difference between the two curves is the endothermic peaks at 287°C that disappeared in the heated SS/SF (50/50) blend nanofibers. There were two weak endothermic peaks at 156°C and 213°C in the SS/SF (50/50) blend nanofibers (Figure 5d); however, there was only one weak endothermic peak at 165°C in the heated SS/SF (50/50) blend nanofibers (Figure 5g).

## Conclusions

We succeeded in producing SS/SF blend nanofibers by electrospinning with a SS/SF TFA blend solution. The "as-spun" nanofibers exhibited smooth surfaces, round cross sections, and bead-free structures. The average diameters of SS/SF (75/25, 50/50, and 25/75) blend nanofibers were thicker than those of SS or SF nanofibers. The mean diameter of these blended nanofibers decreased, and the number of beads slightly increased with increasing dissolving time of the SS/SF blend solution prior to electrospinning. The number of beads also increased with increasing SF content.

The SS/SF (100/0, 75/25, and 50/50) blend nanofibers were easily dissolved in water, while the SS/SF (25/75 and 0/100) blend nanofibers were not completely dissolved in water. The SS/SF blend nanofibers were not dissolved in methanol. From the FTIR measurements, the SS/SF blend nanofibers were shown to possess a random coil conformation and ß-sheet structure. According to FTIR, DSC, and DTA measurements, "as-spun" nanofibers, including SS, SS/SF, and SF nanofibers, contained small amounts of TFA used as the solvent to dissolve the samples. For the SS/SF (50/50) blend nanofibers heat-treated at 160°C, the TFA contained in the sample nanofibers was almost completely removed.

These SS/SF nanofibers are of considerable interest for different kinds of application because these proteins have specific functions, as mentioned in the introduction part, and unique properties including comparatively high specific surface area, etc.

## Competing interests

The authors declare that they have no competing interests.

## Authors' contributions

XZ carried out experimental works on SEM, DSC, FTIR etc. MT participated in the desigin of the study and drafted the manuscript. HM excised efficient supervision of this work. KA carried out the DSC measurement of the samples. GZ participated in the average diameter distribution analysis. MM carried out the statistical analysis of average diameter distribution. All authors read and approved the final manuscript.
